# Travel microbiota: a novel frontier in travel medicine exploring microbial shifts across transportation modes

**DOI:** 10.1186/s40794-026-00292-5

**Published:** 2026-02-10

**Authors:** Pooja Tiwary, Krishil Oswal, Nikolay T. Tzvetkov, Olena Litvinova, Atanas G. Atanasov, Ryan Varghese

**Affiliations:** 1 Department of Pharmacy Practice, Poona College of Pharmacy, Bharati Vidyapeeth (Deemed to be) University, Pune, 411038 India; 2 Department of Biochemical Pharmacology and Drug Design, Institute of Molecular Biology “Roumen Tsanev” Bulgarian Academy of Sciences Sofia, Sofia, Bulgaria; 3 Department of Management, Marketing and Quality Assurance in Pharmacy, National University of Pharmacy of the Ministry of Health of Ukraine, Kharkiv, Ukraine; 4 Ludwig Boltzmann Institute Digital Health and Patient Safety, Medical University of Vienna, Spitalgasse 23, Vienna, 1090 Austria; 5 Department of Biochemistry, Saveetha Medical College and Hospital, Saveetha Institute of Medical and Technical Sciences, Chennai, Tamil Nadu India; 6 Institute of Genetics and Animal Biotechnology of The Polish Academy of Sciences, Jastrzebiec, Magdalenka, 05-552 Poland; 7 Patient Safety and Digital Health (PaDiH) Group, Fakultät Medizin/Zahnmedizin, Danube Private University, Steiner Landstraße 124, Krems-Stein , 3500 Austria; 8 Department of Pharmaceutical Sciences, Philadelphia College of Pharmacy, Saint Joseph’s University, Philadelphia, PA 19104 USA

**Keywords:** Dysbiosis, Gut microbiota, Microbiome, Pathogens, Travel microbiota, Transportation

## Abstract

**Background:**

Between 2010 and 2019, international travel increased by approximately 52.2%, highlighting the world’s dependence on transportation for global connectivity. Although travel enhances global interactions, it also poses risks to public health through the potential transmission of diseases. The rapid global transmission of infectious diseases, exemplified by the outbreaks of COVID-19 and Zika virus, underscores the critical need for in-depth research into travel-associated disease dissemination. When individuals travel, they are exposed to a variety of diverse microbial environments, which can affect their healthy microbiome. In this review, we introduce the concept of “travel microbiota” to encapsulate the dynamic shifts in human microbial communities induced by travel across different transportation modes. This disruption can affect metabolic and immune functions and potentially facilitate the spread of diseases. Given these implications, it is crucial to investigate how different modes of transportation affect the human microbiota. Our study reviews the impact of travel on the human microbiota, highlighting differences across transportation modes. The objective is to establish a framework for understanding travel health and the role of microbiota in managing travel-related health risks. A comprehensive understanding of this relationship is essential for developing preventive strategies to safeguard and restore the human microbiota.

**Methods:**

To provide the specific content, relevant publications were identified on Google Scholar, PubMed, and Science Direct using specific keywords such as dysbiosis, gut, health, microbiome, microbiota, pathogens, travel, and transportation. We did not add any limits to the publication date during the inclusion of papers. However, it is noteworthy that the initial reports, including the aforementioned keywords, have been published starting from 2015.

**Conclusion:**

Travel has a profound impact on the human microbiota, and it is essential to consider the implications associated with various modes of transportation. Traveling through various modes of transportation, such as roadways, airways, and maritime, has significantly influenced human microbiota. Moreover, it acts as a dynamic interface for microbial exchange driving rapid shift in microbial diversity, community convergence, and the diversification of resistant genes. However, the underlying mechanism of these changes remains elusive. By integrating evidence across multiple modes of transportation, this review highlights travel as an underrecognized determinant of microbiome variability and introduces the term “Travel microbiota”. Moreover, this review is pivotal for understanding the ways in which travel alters microbial diversity and developing effective interventions. It is imperative to conduct future research that focuses on conducting large-scale longitudinal studies to assess the effects of traveling on microbial composition and to develop potential preventive measures.

## Introduction

The global volume of international tourist arrivals has risen 52.2%, from approximately 962.56 million in 2010 to 1.465 billion in 2019 [1]. The number of international air passengers has dramatically risen from 0.865 billion to 1.58 billion between 2010 and 2019, highlighting extensive global connectivity [2]. Travelers are subjected to a wide range of health risks based on their destination, mode of transport, duration of stay, and travel behavior, such as dietary choices, hygiene practices, and physical activity [3].

Travel epidemiology serves as a backbone for analyzing the patterns, causes, and health and disease conditions [4]. Moreover, it includes the effects of travel on disease transmission and preventive measures that can aid in the mitigation of these risks [3]. Additionally, travel has been known to disrupt the microbiome of travelers worldwide, often paving the way for the global spread of infectious diseases [5]. The COVID-19 pandemic has significantly affected the spread of travel-related diseases. Prior to the pandemic, 1.58 billion outbound air passengers in 2019 facilitated the swift global transmission of infections [6]. Infectious diseases among travelers entering China were prevalent, particularly among individuals arriving from regions such as Africa, the Americas, and the Western Pacific. However, with the implementation of stringent travel restrictions and lockdowns during the pandemic, commercial flights decreased by nearly 92% in commercial flights between February and April 2020 [7]. This decrease in travel has been positively correlated with disease transmission since March 2020 [8]. As restrictions eased, travel volume gradually increased in certain provinces and territories, although it remained below the pre-pandemic levels [6]. Consequently, there is increased focus on pre-travel consultations, vaccinations, robust surveillance, and quarantine measures [9]. The pandemic has been a testament of the connection between disease transmission and global connectivity, emphasizing the need for public health measures to manage travel-related health risks [10, 11]. Similar results have been reported for the Zika virus, which has been disseminated to new regions worldwide due to travel [12–15].

Travel by land, sea, or air poses significant health risks from bacteria, viruses, and fungi, including ESKAPE pathogens (*Enterococcus faecium*, *Staphylococcus aureus*, *Klebsiella pneumoniae*, *Acinetobacter baumannii*, *Pseudomonas aeruginosa*, and *Enterobacter* species) [16]. Commuters face heightened respiratory tract infections (RTI) risk from airborne microbiota in crowded transport, while sea and air travel increase susceptibility to norovirus, hypoxia-related complications, and infections, including SARS, influenza, and tuberculosis [17–21].

In recent years, increasing attention has been directed toward passenger experiences and microbiome-related data across a wide range of transportation modalities, including rail, terrestrial, maritime, and aviation systems. In light of an exhaustive review of the literature in the aforementioned field, we propose a novel and critically underexplored domain of scientific inquiry: the field of “travel microbiota”. We believe that this conceptual framework delineates the dynamic alterations in the nature of diversity, underlying composition, and the functional capacities of the human microbiota, predominantly precipitating from travel and travel-induced exposures. Unlike isolated descriptions of travel-related dysbiosis or general environmental microbiome exposure, the travel microbiota framework integrates mode-specific environmental factors, microbial ecological dynamics, and host physiological stressors such as circadian disruption, dietary constraint, and confinement into a unified model [22, 23]. Importantly, it underscores the fact that these exposures are shaped by the unique environmental, physiological, and ecological stressors that are inherent to each transportation modality, making them a crucial yet overlooked determinant of health outcomes. This new paradigm identifies travel as an underappreciated environmental stressor capable of perturbing host microbial homeostasis, thereby extending the scope of travel medicine to include microbiome dynamics. By positing both the domain as well as the term “travel microbiota”, we believe in its potential as an emerging frontier in interdisciplinary research. This review underscores the transformative potential of integrating microbiota-centric paradigms as the cornerstone of travel health strategies and clinical practices. The modulation of the human microbiota during travel represents a critical nexus at the intersection of environmental exposures, host physiology, and microbial ecology, offering unique opportunities to advance our understanding of the etiopathology of travel-associated health conditions. These conditions include, but are not limited to, infectious diseases, gastrointestinal disturbances, immune dysregulation, autoimmune disorders, cardiovascular diseases, and stress-related psychological responses.

Furthermore, the emerging field of “travel microbiota” bridges microbiology, travel medicine, environmental sciences, and public health and policy-making, fostering a holistic understanding of the impact of travel-associated perturbations to the microbial ecology at both individual and population levels. By establishing a foundational framework for this nascent discipline, we aim to catalyse further investigations into the mechanistic underpinnings of the triggers, health risks, and resilience mechanisms of the microbiota in travellers. The integration of “travel microbiota” into scientific research delivers significant promise to advance preventive healthcare, refine therapeutic strategies, and improve the safety and experience of travelers. This innovative approach redefines the ill-known relationship between human mobility and microbial ecosystems, serving as the springboard for groundbreaking insights into global health and microbial dynamics in an interconnected world.

## Methodology

This review was conducted in accordance with the Preferred Reporting Items for Systematic Reviews and Meta-Analyses (PRISMA) 2020 guidelines to ensure transparency, reproducibility, and methodological rigor. A structured literature search strategy was employed to identify relevant studies examining the relationship between travel, transportation modalities, and the human microbiome. Relevant publications were identified through comprehensive searches of Google Scholar, PubMed, and ScienceDirect. The search strategy incorporated combinations of predefined keywords, including dysbiosis, gut, health, microbiome, microbiota, pathogens, travel, and transportation. Boolean operators (“AND”/“OR”) were applied to refine the search and capture studies addressing both microbiome-related outcomes and travel-associated exposures. No restrictions were applied regarding publication date to ensure broad coverage of the available literature. Notably, although no date limits were imposed, the earliest studies meeting the inclusion criteria were published from 2015 onward, reflecting the relatively recent emergence of this research domain.

**Fig. 1:**
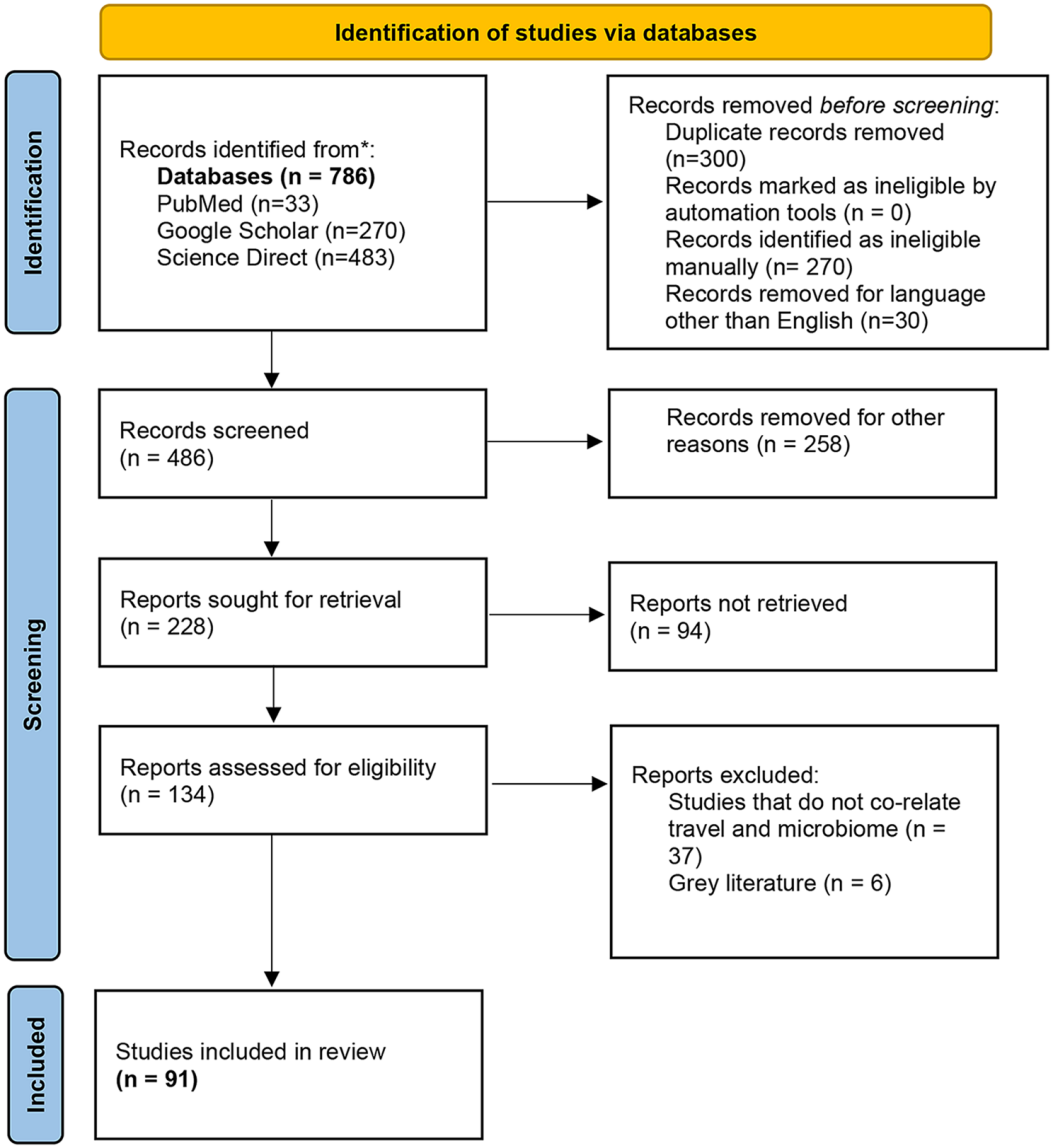
PRISMA flow diagram illustrating the study selection process for the impact of travel on microbiome

All records retrieved from the databases were collated, and duplicate entries were removed prior to screening. Titles and abstracts were initially screened for relevance, followed by full-text assessment of potentially eligible studies. Articles were excluded at the screening and eligibility stages if they did not directly address travel-related microbiome changes or lacked sufficient methodological detail. The study selection process followed the PRISMA 2020 framework and is summarized in the accompanying PRISMA flow diagram (Fig. 1), which details the number of records identified, screened, excluded, and included at each stage.

## The human microbiome

The exploration of the human microbiome, while a relatively recent area of scientific focus, has its roots in the 17th century. Antonie van Leeuwenhoek’s meticulous observations in 1683 revealed substantial diversity within his oral and fecal microbiota, marking a pivotal moment in the field of microbiology. This served as a cornerstone for comprehending the microbial richness in the human body [24]. The pivotal link between microbes and the development of diseases was subsequently unveiled with the groundbreaking revelation of the “germ theory of disease” in the 19th century [25].

Joshua Lederberg coined “microbiome” in 2001, distinguishing it from “microbiota.” While microbiota denotes microbial taxa, the microbiome includes both microbes and their genes [26, 27]. The discovery of the “microbiome” sparked further investigation into the human microbiome and its relationship with the host, leading to the launch of “the human microbiome project” by the National Institutes of Health (NIH) in 2007 [28].

**Fig. 2:**
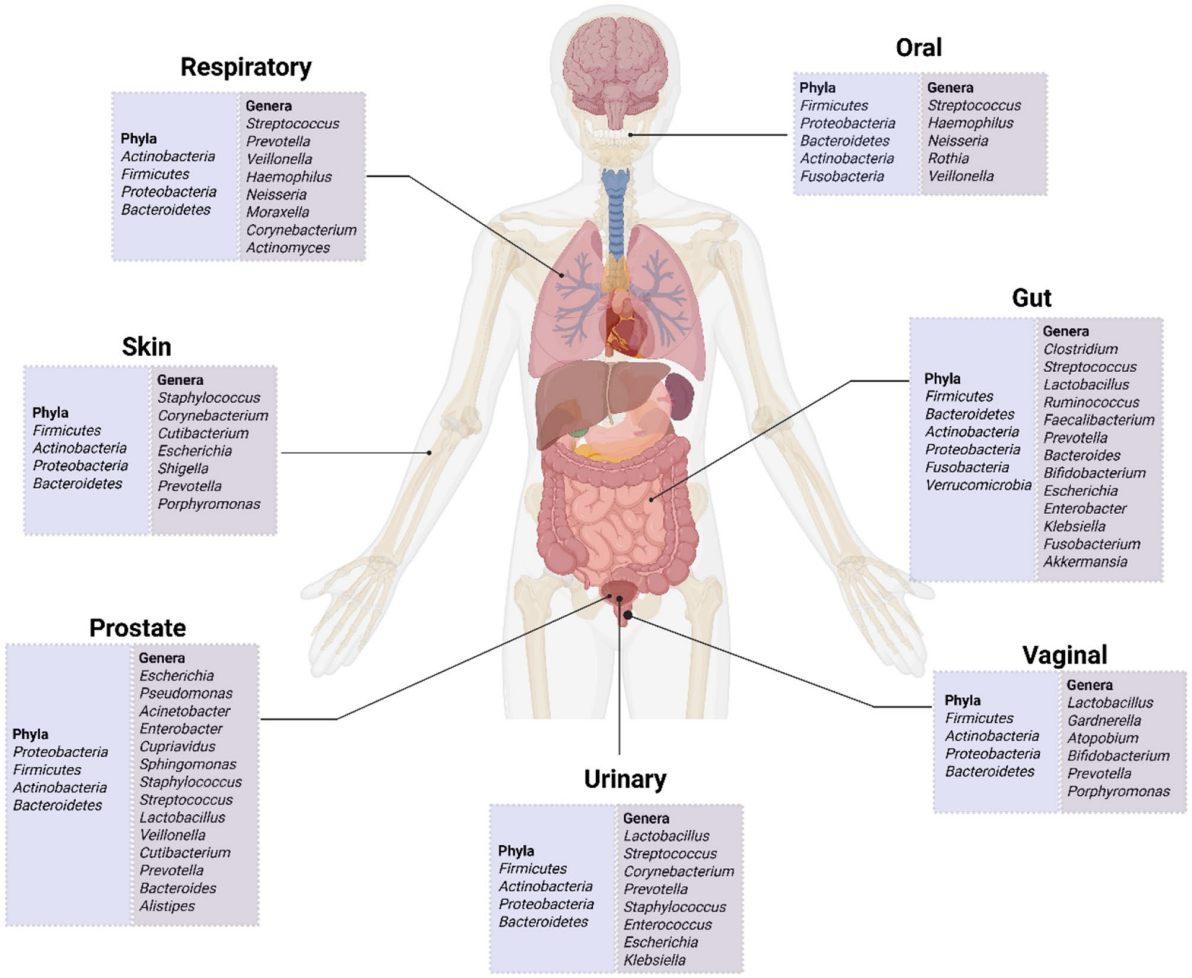
Microbial composition across different human body sites. The schematic illustrates the dominant microbiota of the gut, skin, oral cavity, respiratory tract, and urogenital system. Created in https://BioRender.com

The human microbiota, also known as the hidden organ, contains trillions of microbes residing within a confined environment at distinct bodily sites, such as the gut, oral cavity, skin, respiratory tract, and vagina (in females). The composition of human microbiota at different locations is shown in Fig. 2. Microbial communities are highly personalized and functionalized, depending on their location within the human body [29, 30]. Various host-related, environmental, and lifestyle factors influence their diversity, making it difficult to characterize an individual’s “healthy microbiota” based on taxonomy alone [31]. However, their identical functional profiles can provide insight into the characterization of healthy microbiota. Richness in diversity and microbial genomes, symbiotic microbial-host relationships, possessing a degree of resilience to the external and internal environment, and the ability to reinstate into a healthy functional profile following perturbations are ideal characteristics of a healthy microbiota [32].

Gut microbiota is essential for maintaining host physiology, assisting in nutrient metabolism, immune regulation, pathogenic colonization resistance, xenobiotic and drug metabolism, gut barrier maintenance, and brain health [29, 31–34]. For instance, they ferment indigestible carbohydrates to produce short-chain fatty acids (ScFAs) that provide energy [35], regulate lipids [29], and glucose metabolism [36], and have anti-inflammatory and anti-carcinogenic properties [36, 37]. Moreover, gut microbes biosynthesize vitamin K and B components [29], facilitating the breakdown of the antioxidant [38]. Besides the gut, the skin and oral microbiota also serve distinct functions that are physiologically important for maintaining human health. Oral microbiota protect against infectious agents, maintaining oral balance, and regulating the immune system [39]. The skin microbiota preserves the skin barrier, maintaining skin pH and moisture level, and influences the immune system, thereby preventing the colonization of pathogens [40].

## Introduction to travel microbiota

The transition of the healthy microbiome from a balanced condition to an imbalanced condition is commonly referred to as “dysbiosis.” Dysbiosis specifically entails the depletion of the diversity and functionality of healthy microbiota, resulting in pathogenic colonization and ultimately transforming the state of physiological conditions into a pathological condition [41]. Key contributors to dysbiosis include dietary habits, such as a Western diet or a diet rich in fats/carbohydrates [42]; medications, such as antibiotics and proton pump inhibitors (PPIs) [43]; and host-related factors, such as genetic predisposition and immune system interactions [44]. Additionally, environmental factors such as exposure to pollutants, toxins, and other harmful chemicals [45] and early life factors, including improper nutrition during pregnancy [46] also influences dysbiosis. The shift from a healthy to a disturbed microbiome can manifest as a wide spectrum of medical conditions, including infectious diseases, inflammatory bowel disease, colon cancer, neurological conditions such as Alzheimer’s disease and dementia, metabolic disorders such as type 2 diabetes and obesity, and autoimmune diseases [29, 31, 47, 48] .In the context of travel medicine, transmeridian and long-haul travel have been linked to a range of physical changes and conditions, including jet lag, poor sleep quality, sleep duration [49], cardiovascular diseases [50], hormonal alterations [51], and gastrointestinal issues [52]. Additionally, travel can significantly impact human microbiota, leading to a state of dysbiosis with potential long-term implications. Such travel-induced microbiome perturbations provide the impetus for defining ‘travel microbiota’ as a distinct phenomenon in this review, underscoring the need to study these changes systematically.

The term “travel microbiota” refers to dynamic changes in the diversity and balance of microbial communities in the human body mid- or post-travel, influenced by various modes of transportation, including roadways, airways, maritime, and spaceflights. These shifts predispose individuals to dysbiosis and potentially alter human health. However, the mechanism underlying this microbial transition remains elusive.

Factors such as environmental exposure, physiological stressors, lifestyle modifications, and immune adaptability in response to interactions with new travelers and the environment can be assumed to be likely contributors [53, 54]. Travel-induced changes in the human microbiota can lead to a spectrum of health consequences, ranging from mild digestive discomfort to severe life-threatening conditions. Despite growing interest in this field, significant knowledge gaps remain in understanding how shifts in the microbiota influence microbial communities and impact overall health. Unravelling the mechanisms underlying these alterations is crucial for developing targeted therapeutic strategies that can mitigate health risks and enhance global public health.

### Dynamics of microbial community while traveling

The mode of transportation has a profound impact on the composition of the microbial community owing to changes in geographic relocation, dietary alterations, and environmental stressors. As a result, one of the most commonly affected areas is the gut microbiome, which has experienced a significant shift in functionality and taxonomy. Cheng et al. [55] conclusively investigated the gut microbiome of Chinese volunteers during their six-month stay in Trinidad and Tobago, revealing clear resemblances to local enterotypes. Similarly, Cheung et al. [56] found a substantial increase in the number of antibiotic-resistant bacteria (ARGs) in the gut microbiome of international Chinese travelers, particularly after visiting low- and middle-income countries (LMICs). Parallelly, Peng and colleagues [57] found that 45.6% of participants carried extended-spectrum β-lactamase (ESBL) producing Enterobacteriaceae before travel. Low gut SCFAs and *Actinobacteria* richness increased ESBL-E acquisition risk.

**Fig. 3:**
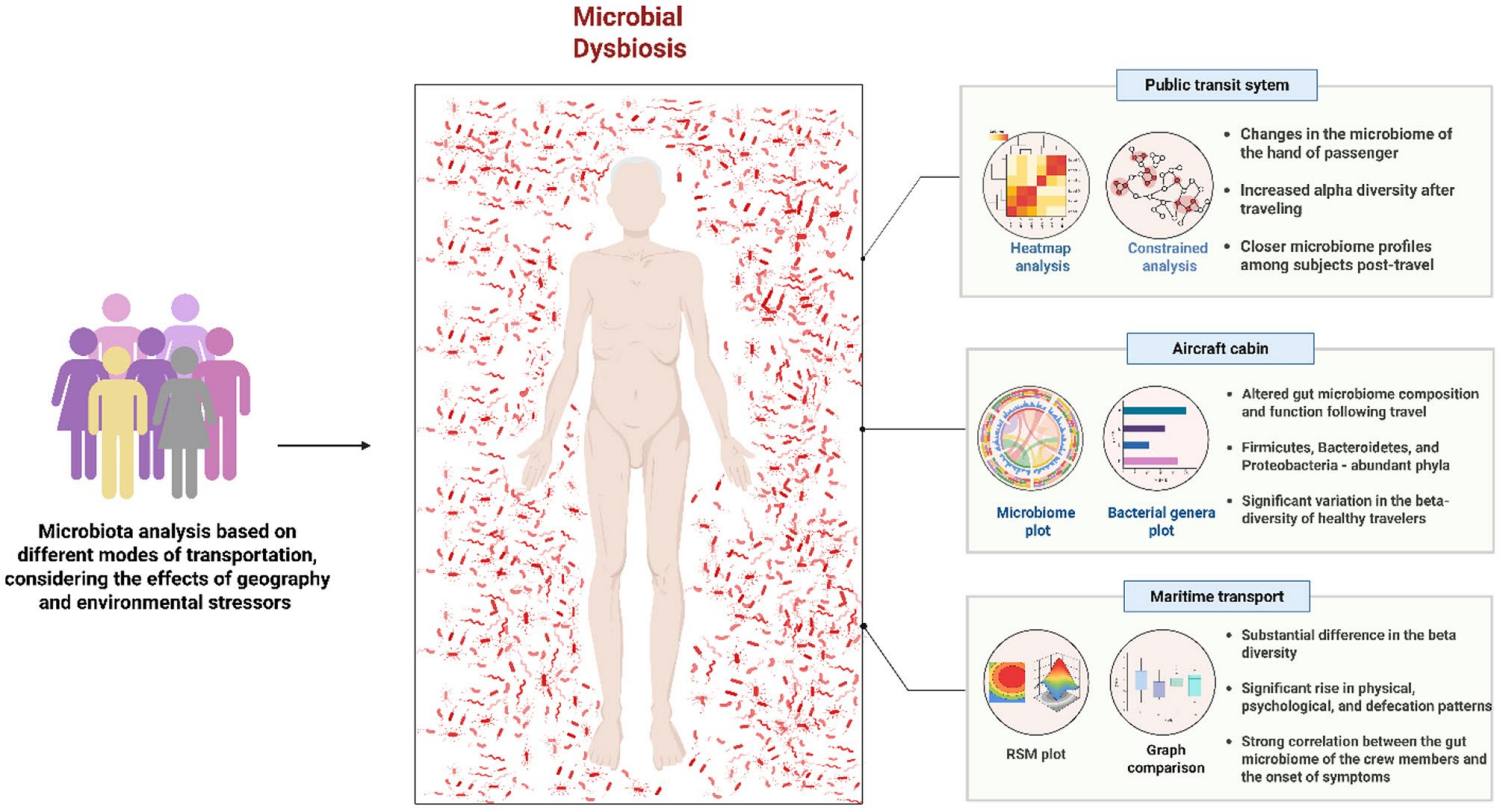
Effect of travel-related microbial dysbiosis across different modes of transportation. The schematic illustrates alterations in human microbiota associated with environmental exposures, stressors, and lifestyle changes during travel by public transit, maritime, and aviation routes. Created in https://BioRender.com

Microbiota analysis across different modes of transportation revealed both shared and mode-specific patterns of microbial shifts (Fig. 3). Across various transportation modes, travel is associated with measurable alterations in gut and hand microbiomes, indicating that movement through distinct environments can perturb human microbial communities. In the public transit users, microbial diversity on the hands increased post-travel, with closer microbiome profiles observed among passengers, suggesting rapid microbial exchange in densely populated settings [58, 59]. Moreover, aircraft travelers exhibited notable changes in gut microbiome composition, particularly in the relative abundance of *Firmicutes*, *Bacteroidetes*, and *Proteobacteria* [60]. Furthermore, there is a significant variation in beta-diversity, reflecting the influence of high altitude and enclosed cabin conditions on the intestinal microbial ecosystem. In addition to this, maritime transport induces substantial beta-diversity shifts and is associated with pronounced changes in physical, psychological, and defecation patterns, indicating a strong interplay between gut microbiota and host physiology in prolonged and isolated travel conditions [61].

Travel affects microbiomes beyond the gut, including oral, respiratory, skin, and urogenital microbiota [53]. Additionally, factors such as temperature, humidity, and exposure to various pathogens during this transition can significantly affect the skin microbiome [62]. Although studies focusing on travel-induced changes in non-gut microbiome are limited. The gut, skin, oral, and respiratory microbiota share common environmental exposures, immune interactions, and microbial transmission pathways. For instance, changes in oral or respiratory microbiota during travel could influence gut microbiome composition indirectly through the gut-lung axis, oral-gut microbial exchange, and systemic immune responses [63]. Similarly, skin microbiome alterations induced by temperature, humidity, or pathogen exposure may modulate host immunity, affecting microbial dynamics at distant sites. Therefore, even in the absence of extensive empirical data, it is plausible to consider an interconnected network of human microbiomes responding collectively to travel-related environmental stressors. These factors collectively impact microbial communities, highlighting the interplay between environment, lifestyle, and microbiota health [63]. We have rigorously classified the alterations in microbiota based on different modes of transportation, considering the effects of geography and environmental stressors during travel, and have elucidated below. This is the first study to establish the impact of various modes of transportation, such as public transit systems, maritime, and aviation, on the adult human microbiota. Through this review, we aim to conclusively demonstrate how traveling and varying environmental interactions affect the normal structure and function of healthy human microbiota, and to uncover strategies for its protection and restoration.

### Microbial dynamics of public transit system

Compared to other modes of transport, the microbiome of public transit stands out as one of the most extensively researched. It is significantly influenced by geographical location, human interaction, environmental exposure, seasonal variation, and the surface within the transit system [64, 65]. Studies on changes in the microbiome while traveling through subways have attracted profound interest. It exposes the human microbiome to various surfaces and air, leading to discernible changes in their microbiota (summarized in Table 1). For instance, a study conducted in Mexico City found that passengers (*n* = 120) interacted with subway surfaces most frequently by hand, resulting in a pronounced exchange of their microbial composition [58]. Moreover, eight passengers were randomly selected to participate in a study examining changes in the microbiome during a subway trip that involved traveling through 11 stations. The experiment included two groups: one group washed their hands beforehand, while the other did not. The group with unwashed hands experienced a slight decrease in the levels of *Acinetobacter*,* Corynebacterium*,* Streptococcus*,* Cutibacterium*, and *Staphylococcus*. Furthermore, the unwashed group recorded an increase in the number of bacterial taxa after traveling. Unwashed hands lost 68.1% of their OTUs and gained 135.1% new ones, whereas washed hands lost 65.3% and acquired 254.4% new OTUs. Unwashed hands retained just 31.9% of their original OTUs, compared to 34.7% for washed hands, which included the most prevalent taxa. The study conclusively discovered that the hand microbiome of passengers significantly increased in diversity after traveling and became strikingly similar to the microbiome of subway.

Another study conducted in the Hong Kong Mass Transit Rail (MTR) system also demonstrated the change in the microbiome upon contact with different handrails for 30 min [59]. The study underscored the paramount importance of intra-day sampling, as clinically significant ARGs increased notably on specific cross-border lines. To illustrate, the TC and ER lines exhibited the highest levels of clinically significant ARGs and showed the greatest potential for community-wide dissemination. The ER line specifically identified five distinct families of signature ARGs. Among these, four (tetA, tetO, tetRRPP, and tetMWOS) conferred resistance to tetracycline, while one (vanC) conferred resistance to vancomycin. Notably, these ARG abundances were most pronounced in the mornings. Subsequently, all lines showed an increase in these ARG families, and by afternoon, they became universally identified signature families.

Our thorough analysis of studies on the impact of public transit systems on human microbiota reveals that microbial communities on transit surfaces are extensively exchanged through contact. The subway environment, in particular, shows significant microbial diversity and acts as a major medium for microbial transmission. Research conducted at a subway station in Bangkok found a high prevalence of bacteria carrying ARGs and xenobiotic degradation genes (XDGs) across air (72%), handrails (56%), and floors (49%) [66]. Notably, *Moraxella osloensis* was the predominant across all sites. Furthermore, floor samples contained bacteria with ARGs that confer resistance to MLS (macrolides, lincosamides, and streptogramins), and the air contained glycopeptide and fluoroquinolone ARGs. The main contributors of ARGs on the floor and handrail were *Klebsiella aerogenes*, while the air contained *Staphylococcus* and *Bacillus* species. The presence of ARGs and the diversity of microorganisms are likely linked to the high density of passenger traffic, close contact, and frequent interactions with subway surfaces, which may contribute to multidrug-resistant (MDR) bacteria. From a travel medicine perspective, the rapid redistribution and presence of ARGs within public transit system carry important public and clinical health implications. Public transit-associated ARGs can persist on frequently touched surfaces and shared environments facilitating repeated exposures or sustained colonization of travelers. Such colonization may not immediately result into illness but can increase the risk of opportunistic infections, thereby reducing the effectiveness of first-line antibiotics. Notably, travelers may acts as vectors, acquiring ARGs-harbouring microbes during transit subsequently introducing them into households, healthcare settings, and or new travel destinations.

In summary, public transit systems function as dynamic hubs of microbial exchange, leading to significant alterations in travellers’ microbiota and facilitating the spread of antimicrobial resistance genes. The studies across multiple transit system reveal consistent pattern of microbial exchange shared space, human mobility, and surface contact. Despite infrastructural and geographic differences, subway environment exhibits high microbial diversity and enrichment of ARGs, all of which directly influences the passenger microbiota. These findings position public transit system as dynamic microbial hubs that not only reshape traveler microbiota but also serves as reservoir and conduits for ARGs. This highlights the need for targeted hygiene intervention, probiotic supplementation, and improved surface materials aimed at mitigating microbial and resistant genes transmission in high-density urban transit network.

**Table 1:** Comparative analysis of microbial dysbiosis findings in public transit system

S. *N*.	Mode of transport	Specific aim of the paper	Demographic characteristics	Research Methodology	Analytical Methodology	Key results	Reported advantages	Reported limitations	Suggestions for further research	Ref.
1.	Subway	To design a longitudinal metagenomic map of mass transit systems and public spaces (New York, Boston, and Sacramento)	Swab samples from the people of 3 cities	Illumina next-generation sequencing of swab DNA samples Bioinformatics clustering	Statistical analyses (ANOVA), PCAs, and network analysis for identification of city-specific microbiome fingerprints	Similarities in the microbiome of passengers’ hands and subway surfaces Microbial richness and diversity reduced after pole cleaning	Dissemination of interchange of microorganisms between passengers and subway surface	Lack of generalizability to other environments	• Study subway microbiome’s role in disease spread and urban microbial control • Investigate the long-term effects of microbial sharing • Comparison of subway microbiomes worldwide for unique and shared microbial patterns	[58]
2.	Train	To study microbial communities on transit surfaces to understand microbial transmission between humans and built environment in mass transit systems.	The study was conducted in the Boston Metropolitan Transit System (238 million trips/year)	Swab samples were collected from the public transit system from surfaces (touchscreens, poles, and ticket machines)	16S amplicon and shotgun metagenomic sequencing	Human skin and oral commensals on transit surfaces Outdoor touchscreens: abundance of *alphaproteobacteria* Functional profiling: enrichment of *Propionibacterium acnes* pathways on train holding surfaces and electron transport chain components on touchscreens and seats.	First high-precision microbial survey in a mass transit system, provides insights into microbial transmission dynamics between humans and built environments in public spaces	-	• Characterization of microbial profiles in multiple transit systems to enhance biosurveillance for ARGs or pathogens • Study on impact of human contact, materials, and environment on microbial profile	[64]
3.	Metro	To determine the microbial diversity profile in Mexico metro system due to its high passenger traffic and microbial transmission	Mexico City population which has 4 million daily users	Swab samples from various surfaces within the metro station	16S rRNA gene sequencing	Distinct and higher microbial diversity in metro stations than in trains	Provides insight into microbial diversity within a high-traffic public transport system	Sampling limitations, impact of external environment on microbial composition	• To test the geographical and host effects in the transport system-associated microbial diversity • Study of species pool transmission and DNA resilience in the metro station will aid in understanding the human microbiome interactions	[67]
4.	Subway	To investigate the microbial diversity within the urban subway network in Hong Kong	People from multiple subway lines in Hong Kong	Aerosols sampling	Culture-independent approach targeting the 16S rRNA gene V4 region	Extensive taxonomic diversity with a prevalence of skin-related genera, bacterial diversity varied based on day, time, and peak vs. non-peak commuting hours	-	Lack of broader study and sample	• Study of mechanism that shape subway microbiome for better understanding of microbial exposure around the globe	[68]
5.	Subway	To determine the microbial diversity and bioaerosols in the population of New York subway environment	Sample from seven subway in New York	Bioaerosols collection through fluid impingement over 1.5 years	Characterization of nano and micron-sized air-borne particles using a multi-analytical approach BLAST analysis for determination of the source of DNA Sanger and pyrosequencing technologies for small subunit rRNA gene sequencing	Simple bacterial composition with identifiable bacterial sequencing composed of a mixture of genera and species from soil, water, and human skin commensal bacteria	Use of culture-independent phylogenetic analysis	-	-	[69]
6.	Mass transit railways (MTR) system	To profile the human palm microbiome after contact with handrails to understand the variation and recurrence in microbial community composition	Human participants who came in contact with the handrails of the MTR system in Hong Kong	Intraday samplings	Metagenomics for analysis of microbial signatures (human commensals, clinical ARGs, and line-specific environmental exposure) Taxonomic profiling, phylogeny, pan-genome analysis, reference-based strain mapping, dissemination potential estimation, Simpson diversity index, statistical analysis using R	Intraday time was the primary determinant of metro microbiome and resistome composition Higher abundance of clinical ARGs and human commensals in p.m. samples	Identification of primary determinant of variation in microbial community composition across the MTR system	Lack of generalizability in the study	• Future studies involving more sampling points to thoroughly describe the intraday dynamics, temporal changes and public health strategies	[59]
7.	Subway	To investigate the microbial profile at a city-wide scale	Sample on human DNA focusing on New York city (NYC) subway, Gowanus canal, and public parks	DNA sequencing	Ancestry Mapper and Admixture tools were used to analyze the human DNA from subway surfaces	Half of the DNA on subway surface did not match any known organism Identification of 1,688 bacterial, viral, archaeal, and eukaryotic taxa enriched for skin-associated genera like Acinetobacter Bacterial signature matched marine-associated bacteria in a flooded station	Potential for long-term monitoring and bioterrorism preparedness	Lack of generalizability	• Long-term studies on changes in microbial communities over time in urban environment could aid in providing valuable insights into disease surveillance and management • Further studies on expansion of metagenomic mapping to other cities for comparison of urban microbial diversity	[70]

### Microbial dynamics of maritime transport

Long-duration Sea voyages expose seafarers to a variety of environmental and occupational stressors. These include high humidity, significant UV radiation, high salinity, confined spaces, stormy seas, varying climate zones, biohazards, chemical exposure, sleep deprivation, and disruptions in circadian rhythms [71–73]. Such harsh conditions can lead to numerous medical issues for crew members, including gastrointestinal disorders [74] and oral diseases [75], musculoskeletal disorders [74], mental health issues [74] and cardiovascular diseases [61]. Collectively, these conditions can severely affect physical and psychological well-being. Furthermore, the cumulative effects of these stressors have been shown to affect the human microbiome, with evidence suggesting that prolonged exposure can lead to significant changes in the microbial composition.

Jiang et al. [76] investigated the effects of a 6-month sea voyage on the gut microbiota of 30 healthy male participants. Fecal samples were collected from all individuals pre- (day 0) and post-voyage (day 180) for 16S rRNA gene sequencing and LC-MS untargeted metabolomics analysis. While the alpha diversity remained largely unchanged, significant alterations in beta diversity were observed in the fecal samples. Species such as *Coriobacteriaceae_UCG-002*,* Bilophila*,* and Faecalitalea* dominated during the start of the voyage (day 0), while *Holdemanella*,* Plesiomonas*,* Weissella*,* and Tyzzerella_3* were among the high-abundance species towards the end of the voyage (day 180). The presence of *Holdemanella* is generally associated with an increased risk of anxiety, depression, and obsessive-compulsive disorder, whereas a negative correlation was observed with 2-hydroxycinnamic acid, which may contribute to the incidence of neuropsychiatric disorders. Similarly, *Plesiomonas*, a pathogen, was positively correlated with acetylcholine, suggesting a potential pathological mechanism for inducing bacterial gastroenteritis. The study also highlighted the potential negative impact of decreased *Holdemanella*,* Bacteroides*,* and Ruminococcaceae_UCG-008* diversity on various human metabolic pathways, such as human protein absorption and digestion and phenylalanine metabolism, hinting at a dysbiotic gut environment post-sea voyage [76].

Furthermore, Seafarers reported significant changes in defecation patterns after returning from voyages, with a nearly 50% increase in the frequency of abnormal bowel movements compared to baseline. Post-travel samples revealed gut microbiome perturbations, indicating that gut dysbiosis is a key factor in the development of seafaring syndrome (SS) [77]. Stool samples were collected at two different time points: day 1 and day 135 after the voyage. The post-voyage samples indicated disruptions in the gut microbiome, suggesting that gut dysbiosis is a key factor in developing SS. Analysis of the changes throughout the voyage revealed a striking 89.5% variation (*n* = 17), underscoring strong associations between microbes, SS, and the development of chronic diseases [77]. Another study by Zheng et al. [78] reported reduced microbial functional diversity, pathogenic colonization, and altered carbohydrate, amino acid, and lipid metabolism, indicating a state of oral and skin dysbiosis among seafarers. The alterations in the microbiota following sea voyage are presented in Table 2. In summary, extended maritime voyages under harsh conditions can profoundly disrupt the gut and oral microbiota of seafarers, contributing to dysbiosis-related health issues observed upon return, such as seafaring syndrome.

**Table 2:** Comparative analysis of microbial dysbiosis findings in maritime transport

S. *N*.	Mode of transport	Specific aim of the paper	Demographic characteristics	Research Methodology	Analytical Methodology	Key results	Reported advantages	Reported limitations	Suggestions for further research	Ref
1.	Ship	To investigate the changes in the gut microbe-host interplay of seafarers and propose potential targets during a 6-month sea voyage	30 healthy male participants (18–35 years) with no reported data on co-morbid conditions	Fresh stool samples were collected using sterile sampling tubes pre- and post-voyage.	Gut-microbiota evaluation − 16S rRNA gene sequencing. Faecal untargeted metabolomics analysis - liquid chromatography-mass spectrometry (LC-MS). Relationships between gut microbiome, faecal metabolites, and human metabolic pathways - Spearman correlation analysis	No significant changes in the alpha diversity Substantial difference in beta diversity High abundance: *Coriobacteriaceae_UCG-002*,* Bilophila*, and *Faecalitalea* at day 0 *Holdemanella*,* Plesiomonas*,* Weissella*,* and Tyzzerella_3* at day 180 Substantial rise in *HIMB11*,* Ramlibacter*,* Finegoldia*,* and Ruminococcaceae_UCG-008* Significant decrease in *Erysipelatoclostridium*,* Bilophila*,* Faecalitalea*,* and Coriobacteriaceae_UCG-002* after the voyage. 14 differential microbiota members strongly associated with 6 differential faecal metabolites.	Multi-omics (microbiomics and metabolomics) approach generated results	Limitation on other microorganisms (viruses, bacteriophages, yeasts, fungi) Failure in establishment of direct causality among the differential microbes, and altered human metabolic pathways	• Analysis of multiple psychological and physiological scales, conducting in-vitro studies to verify the underlying causality among long-term voyages, physical and psychological disorders, and the gut microbiome/metabolome	[76]
2	Ship	To evaluate the association between gut microbiome and the health status of the crew members during a 5-month (135 days) long ocean voyage	77 male Chinese crew members (20–35 years)	During the voyage, crew members were subjected to a questionnaire (physical, psychological, and defecation-related indicators): scoring-based system employed for categorization into healthy and symptomatic groups	Whole metagenome sequencing (WMS) - to assess the relationship between gut microbiome and health status of the crew members	Significant rise in physical symptoms (backache, headache, muscular soreness, and stomach ache), psychological symptoms (poor sleep quality, insomnia, and overeating), and defecation patterns (abnormal frequency of defecation, incomplete defecation and/or constipation). Strong correlation between the gut microbiome of the crew members and the onset of symptoms. Crew members suffering from seafaring syndrome (SS) displayed perturbations in the gut microbiome between the two-time frames (day 1 and day 135). In total, 19 microbial species were found to be altered in the SS group during the voyage. Out of which, 17 had a previous correlation with chronic diseases	-	-		[77]
3	Ship	To investigate the impact of long sea voyages on the microbial communities in the sailors’ oral mucosa and belly buttons and their correlation with overall health.		Specimen collections from the oral cavity in distinct locations (saliva, soft tissues, supra- and subgingival dental plaque) Sampling of the belly button was carried out using swabbing	Combination of 16S rRNA sequencing and whole genome shotgun sequencing (WGS) - to characterise the microbial diversity during the long sea voyage	Significant 5-fold reduction in the alpha diversity of buccal mucosal microbes and a two-fold reduction in belly button microbial diversity after the voyage Significant increase and decrease in *Firmicutes and Proteobacteria*, respectively, post voyage. Insignificant rise in *Streptococcus* and *Staphylococcus* WGS sequencing revealed a 99.97% composition of the total bacteria, out of which 69.46% were unclassified Rise in the level of opportunistic pathogens after the sea voyage was also reported. The Taxonomic analysis of the 16S rRNA sequencing in the belly button microbiota revealed a significant increase in *Firmicutes*,* Staphylococcus* and a subsequent decline in *Corynebacterium* Significant drop in functional genes after the sea voyage, including three major metabolic pathways involving carbohydrates, lipids, and amino acids Significant decrease in microbial folate synthesis and inadequate administration of fresh fruits and vegetables and vitamin supplements compromised the serum folic acid levels of the sailors Serum concentration of homocysteine (HCY) showed an increase after the sea voyage with no significant change in the serum vitamin B12	-	Influence of microbial metabolites on host biology. Sample size	-	[78]
4	Ship	To investigate the functional relationship between oral microbiota and sailors’ health among ocean voyagers	12 healthy males (22–55 years)	Passive saliva samples were collected, frozen	16S rRNA sequencing and whole genome shotgun sequencing (WGS) - to illustrate the microbial diversity.	Increase in alpha diversity during the 25 days of the voyage Substantial increase in *Proteobacteria*,* Bacteriodetes*, and *Fusobacteria* after 25 days of the voyage Significant rise in *Enterococcus*,* Pseudomonas* and *Lactobacillus*	-	-	Further research aiming to increase the sample size population should be considered.	[79]

### Microbial dynamics of aircraft cabins

The microbiome of airplane cabins demonstrates substantial variability across flights, and is highly individualized and characterized by distinct microbial communities. The composition of the cabin microbiome contains remnants of its past microbial life, that is, on-board passengers. While it has been found that most microbes are not harmful [80], some studies have reported the presence of virulent factors and chemicals that could contribute to the likelihood of infectious and inflammatory diseases [81]. Multiple factors, such as environmental conditions, dietary changes, flight duration, and other confounding factors, may play a role in inducing a dysbiotic state. For instance, mild hypoxia, low humidity, and an environment conducive to pathogenic microbial colonization can contribute to dysbiosis [82]. Additionally, factors such as the consumption of processed or fatty foods [83] and disruptions in circadian rhythms, sleep patterns, and jet lag may also contribute to dysbiosis [84]. However, the exact mechanisms underlying these relationships have not yet been well established and require further investigation.

A study conducted by Venable et al. [85] found that air travel has a significant negative impact on the gut microbiota of canines. The dogs were randomly divided into two groups: control and travel. Dogs in the control group were housed individually, whereas those in the travel group were flown in the cabin of an airline for a flight lasting 2.5 h. After flight, they were taken to a search site and housed in temperature-controlled kennels. Blood and fecal samples were collected from both groups before flight and search, respectively. Beta diversity analyses revealed significant changes in the bacterial communities of the travel group (*p* = 0.01) compared with the control group, as well as significant differences in bacterial community abundance (*p* = 0.02). Specifically, *Clostridia* class (67.78% versus 50.99%) and *Bacteroidaceae* family (6.43% versus 1.09%) were found to be significantly higher in the travel group than in the control group. Additionally, there was an increase in the relative abundance of *Bifidobacteriaceae* (*p* = 0.03) from day 0 to day 3, from 0% to 0.23%. The *Blautia* genus also increased (*p* = 0.05) from 8.99% on day 0 to 15.02% on day 3 in the travel group.

Another study analyzing the gut microbiota of an airplane pilot showed a significant decrease in health-promoting bacteria [86]. Furthermore, analysis of the gut microbiome of Irish cricketers after travel to India revealed disruptions in the gut microbiota [60]. Moreover, the development of travelers’ diarrhea was further associated with air travel-related dysbiosis [54]. These findings (summarized in Table 3) suggest a direct association between air travel and dysbiosis. However, further in-depth investigations are required to fully understand the impact of air travel on the gut microbiome. In summary, the unique conditions of air travel—confined cabins, low humidity, mild hypoxia, and circadian disruption—can acutely perturb the gut microbiome, although the reversibility and clinical significance of these changes remain to be fully determined.

**Table 3:** Comparative analysis of microbial dysbiosis findings in aircraft cabin

S. *N*.	Mode of transport	Specific aim of the paper	Demographic characteristics	Research Methodology	Analytical Methodology	Key results	Reported advantages	Reported limitations	Suggestions for further research	Ref
1.	Aeroplane	To investigate the impact of travel on gut microbiota fluctuations in Irish cricketers.	14 male cricketers and 7 female cricketers were included in this study	Pre- and post-travel faecal samples were collected from male and female cricketers.	16S rRNA sequencing was employed for all samples. Additionally, for a subset of samples from 4 male cricketers who provided samples at all 6 time points, shotgun metagenomic sequencing was employed.	Travel to India altered the gut microbiome composition and function, increased antibiotic resistance genes and virulence genes Significant increase in the *E. coli* species among 2 individuals following travel to India. 2 players acquired antibiotic resistance-related genes. Additionally, the virulence marker genes were significantly increased post-travel	-	-	-	[60]
2	Aeroplane	To investigate the association between bacterial gut communities and traveller’s diarrhoea	A total of 111 participants (99 developed traveller’s diarrhoea and remaining 12 remained healthy)	Stool samples	16S rDNA sequence analysis was employed.	*Firmicutes*,* Bacteroidetes*, and *Proteobacteria* - abundant phyla in the diarrheal groups High *Firmicutes: Bacteroidetes* ratios were observed, resembling a dysbiotic gut microbiome of diarrhoeal groups Significant variation in the beta-diversity of healthy travellers and pathogen-associated diarrhoeal group Comparison of healthy traveller microbiota to those of a healthy person who were actively involved in the HMP project - healthy subjects possess significant *Firmicutes: Bacteriodetes* ratio coupled with greater beta-diversity.	-	-	This study emphasize need for further research that will identify the mechanistic relationship between travel, gut microbiome, ad development of diarrhoea	[54]

## Conclusion and outlook

Our comprehensive evaluation highlights the profound impact of travel on the human microbiota, offering critical insights into how different modes of transportation lead to shifts in microbial diversity. While these findings enhance our understanding of travel-induced microbiome alterations, their long-term consequences remain largely uncharted. Notably, some evidence suggests that many travel-induced microbiome changes may be transient, with the microbiota gradually reverting toward its pre-travel state once travel concludes. Addressing this gap requires rigorous, multidisciplinary research to elucidate the lasting effects of microbiota perturbations and their potential implications for human health. Moreover, it is unclear whether the microbiota perturbations observed during travel are causative drivers of post-travel illnesses or merely correlates of travel-related stressors, highlighting the need for longitudinal studies to establish causality.

A key priority for future research is the development of targeted interventions to mitigate potential adverse effects and promote microbiome stability. Promising strategies, such as colonic probiotic insufflation [87], tailored probiotic supplementation, and dietary modifications, warrant further investigation in well-designed clinical trials. Additionally, integrating microbiome restoration techniques into personalized travel health strategies could offer a proactive approach to maintaining the gut microbial balance.

A key limitation of the current evidence base is that many studies examining travel-associated microbiome change involve small sample sizes, animal models, and highly specific populations. While these studies provide valuable proofs-of-concept and mechanistic insights, their finding may not be fully generalizable to the broader traveling population, which encompasses diverse ages, health statuses, travel purposes, and exposure profiles. Moreover, controlled and occupational settings may amplify the stressors, such as dietary restrictions, confinement, or environmental exposure, potentially exaggerating microbiome effects compared with routine civilian travel. Additionally, the effects of key travel-related factors such as transportation modality, destination-specific environmental exposure, hygiene practices, stressors, circadian disruption, and antibiotic use are highly confounded. As these variables frequently co-occur during travel, isolating the contribution of any single factor remains challenging in most observational studies. Consequently, the associations described in this review should be interpreted with caution, as they reflect a composite exposure effect rather than a discrete causal mechanism. The current limitations in the scope of this study and transportation modality coverage highlight the need for broader, globally representative research. Future studies should assess microbiota shifts across diverse public transportation systems, accounting for region-specific microbial exposure. Given the increasing reliance on air travel, large-scale, multicenter studies focusing on microbial changes in airline passengers are particularly critical. Understanding the influence of cabin air filtration, altitude shifts, and prolonged exposure to confined environments will help delineate their roles in shaping microbiome dynamics.

Future research should focus on delineating dietary and probiotic strategies to mitigate microbiome disturbances during travel, particularly in contexts of limited dietary diversity such as long-haul flights, sea voyages, and space missions. While high-fiber diets show potential for microbiome remodeling through altered metabolic capacity, longer intervention durations and integrative approaches are required to confirm benefits. Fermented foods demonstrate stronger effects on microbial diversity and inflammation, warranting controlled trials in travel-associated stress conditions [88]. Probiotics, especially *Saccharomyces boulardii* and *Lactobacillus rhamnosus* GG, have shown promise in reducing travelers’ diarrhea and maintaining microbiome homeostasis, though strain-specific efficacy requires clarification [89, 90]. The enrichment of carbohydrate-active enzyme–encoding taxa with probiotics further supports their role in sustaining metabolic resilience [91]. Travel medicine practitioners should consider microbiome-preserving interventions in their advice to travelers—for example, recommending appropriate probiotic/prebiotic supplementation and discouraging unnecessary antibiotic use—to help maintain travellers’ gut health during and after travel [92]. Future investigations should draw on insights from latest biotechnology advances and integrate dietary, probiotic, and environmental variables to establish personalized strategies for microbiome modulation [47, 93], ultimately enhancing traveler health and performance in constrained or extreme environments. The microbiota targeted intervention may be particularly beneficial for specific particular populations associated with heightened microbial disruptions. These include long-haul air travelers, maritime personnel, subway passengers, frequent international travelers, immunocompromised individuals, older adults, and individuals with pre-existing gastrointestinal and metabolic disorders. Such individuals are exposed to dietary monotony, prolonged confinement, circadian misalignment, psychological stress, and increased risk of antimicrobial resistance, all of which can exacerbate microbiome instability.

Moreover, leveraging advanced methodologies, such as bioengineering, functional metagenomics screening, bioinformatic pipelines, metagenomics, meta-omics, machine learning, and AI-driven predictive analytics, can revolutionize real-time microbiome assessments. These innovations will enable deeper insights into microbiota alterations and facilitate the development of precision-based interventions tailored to the unique microbiome needs of travelers. By bridging the existing knowledge gaps and integrating cutting-edge scientific advancements, future research can pave the way for novel strategies to preserve microbiome integrity during travel. Equally critical is a robust public health framework that incorporates travel microbiota surveillance into global disease monitoring, in order to detect and mitigate the international spread of antimicrobial resistance or novel pathogens via travelers at an early stage. Ultimately, these efforts will not only enhance traveler health but also contribute to a broader understanding of microbial interactions in transient populations, reinforcing global public health initiatives.

## Data Availability

No datasets were generated or analysed during the current study.

## Declarations

### Name of the registry

Not required. [Please note that this is a Review article which provides a comprehensive landscape of microbial dysbiosis during traveling and introduces the concept of travel microbiota. It is not a research study/ clinical trial/systematic review, and therefore does not require any Registry number].

### Unique identifying number or registration ID

Not applicable/Not required.

### Hyperlink to your specific registration (must be publicly accessible and will be checked)

Not applicable/Not required.

### Guarantor

Ryan Varghese (Corresponding author) and Atanas G. Atanasov (Corresponding author), and Olena Litvinova (Corresponding author).

### Data statement

The data in this Review article is not sensitive in nature and is accessible in the public domain. The data is therefore available and not of a confidential nature.

### Provenance and peer review

Not commissioned, internally peer-reviewed.
